# Supplementary figures and images for: Protein kinase C in the wood frog, Rana sylvatica: reassessing the tissue-specific regulation of PKC isozymes during freezing
Source: PeerJ. 2014 Sep 4;2:e558. doi: 10.7717/peerj.558 (PMC4157297; doi:10.7717/peerj.558)

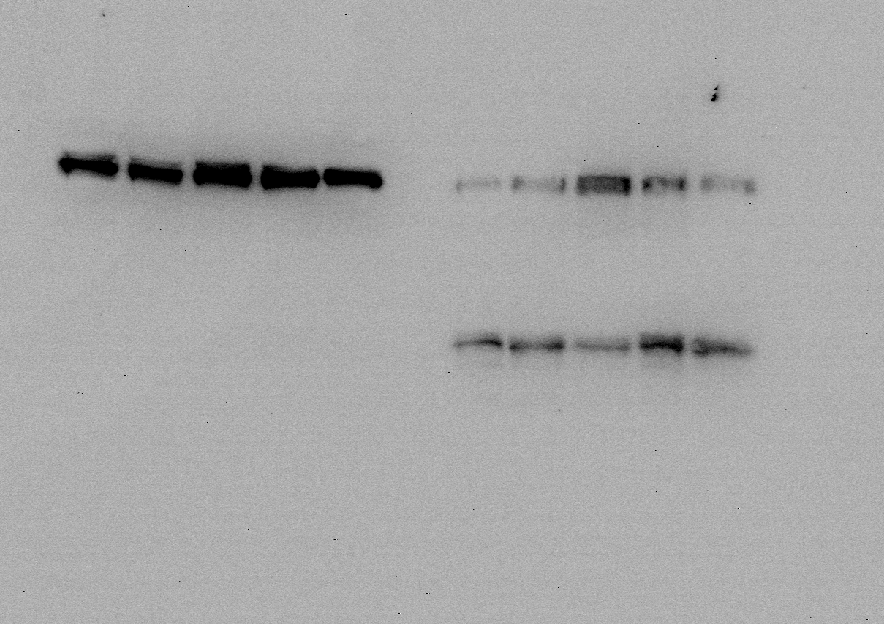

Supplement: Supplemental Information 1 — A full blot is presented here for liver, but in other instances PVDF membranes were typically cut at the approximate molecular weight of PKC so that membranes could be used for multiple antibodies (as described in Materials and Methods). 5 independently-prepared protein extracts from control frogs (left) and 5 independently-prepared protein extracts from frozen frogs (right) were electrophoresed, transferred, immunoblotted, and exposed in parallel. [file peerj-02-558-s001.png]

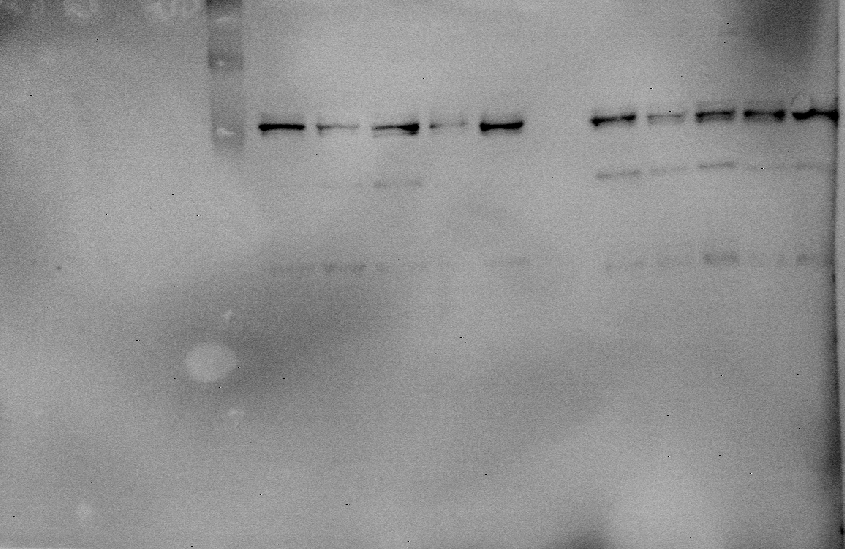

Supplement: Supplemental Information 2 — A full blot is presented here for kidney, but in other instances PVDF membranes were typically cut at the approximate molecular weight of PKC so that membranes could be used for multiple antibodies (as described in Materials and Methods). 5 independently-prepared protein extracts from control frogs (left) and 5 independently-prepared protein extracts from frozen frogs (right) were electrophoresed, transferred, immunoblotted, and exposed in parallel. [file peerj-02-558-s002.png]

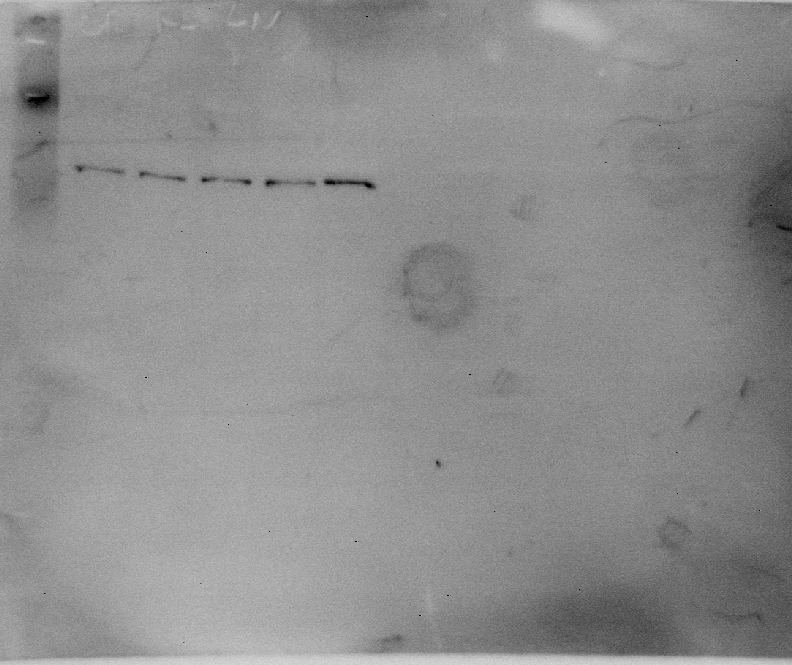

Supplement: Supplemental Information 3 — A full blot is presented here for liver, but in other instances PVDF membranes were typically cut at the approximate molecular weight of PKC so that membranes could be used for multiple antibodies (as described in Materials and Methods). 5 independently-prepared protein extracts from control frogs (left) and 5 independently-prepared protein extracts from frozen frogs (right) were electrophoresed, transferred, immunoblotted, and exposed in parallel. [file peerj-02-558-s003.png]

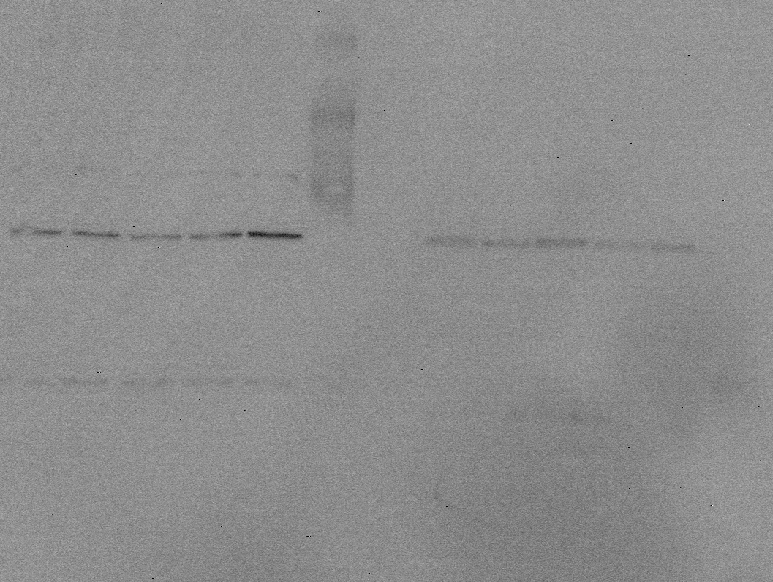

Supplement: Supplemental Information 4 — A full blot is presented here for liver, but in other instances PVDF membranes were typically cut at the approximate molecular weight of PKC so that membranes could be used for multiple antibodies (as described in Materials and Methods). 5 independently-prepared protein extracts from control frogs (left) and 5 independently-prepared protein extracts from frozen frogs (right) were electrophoresed, transferred, immunoblotted, and exposed in parallel. [file peerj-02-558-s004.png]

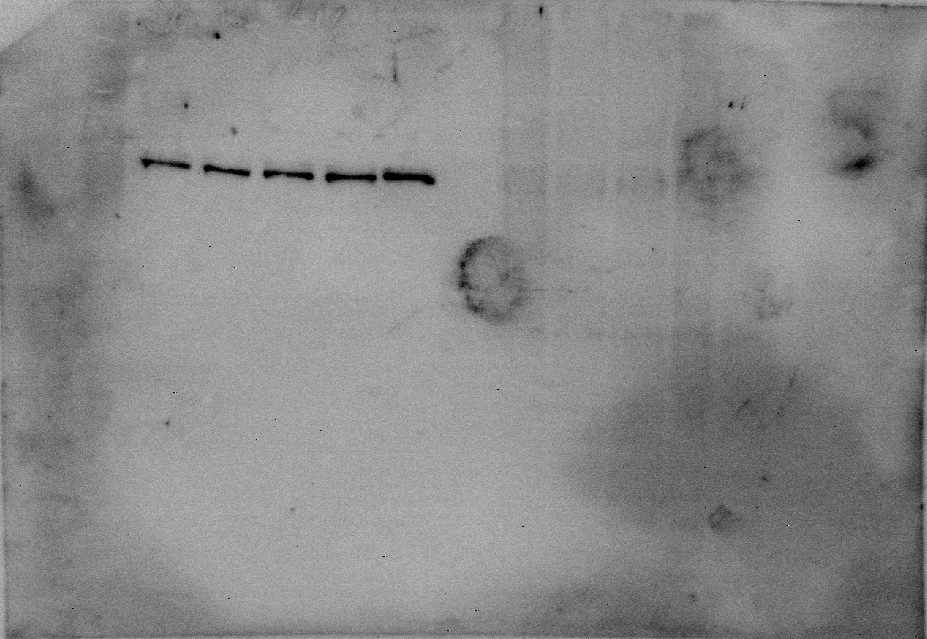

Supplement: Supplemental Information 5 — A full blot is presented here for liver, but in other instances PVDF membranes were typically cut at the approximate molecular weight of PKC so that membranes could be used for multiple antibodies (as described in Materials and Methods). 5 independently-prepared protein extracts from control frogs (left) and 5 independently-prepared protein extracts from frozen frogs (right) were electrophoresed, transferred, immunoblotted, and exposed in parallel. [file peerj-02-558-s005.png]

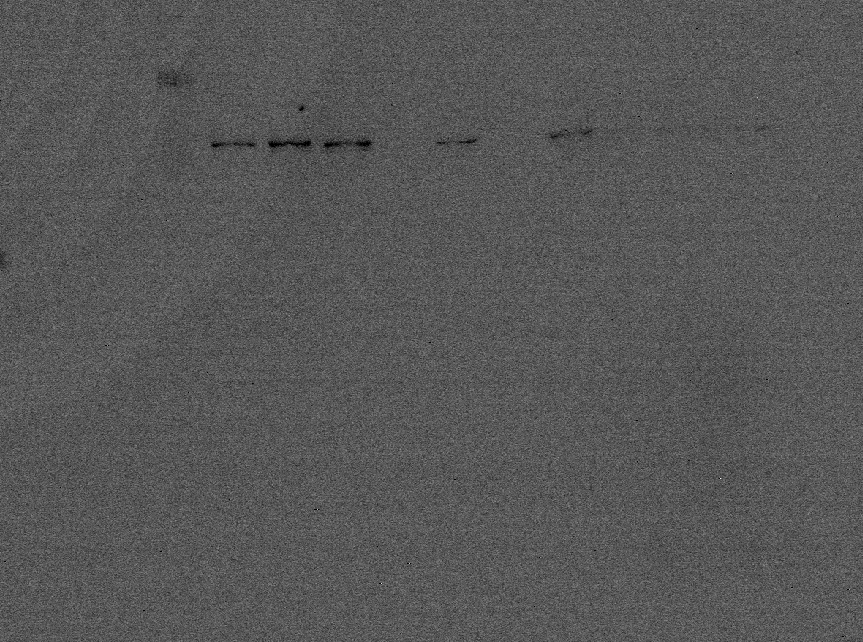

Supplement: Supplemental Information 6 — A full blot is presented here for kidney, but in other instances PVDF membranes were typically cut at the approximate molecular weight of PKC so that membranes could be used for multiple antibodies (as described in Materials and Methods). 5 independently-prepared protein extracts from control frogs (left) and 5 independently-prepared protein extracts from frozen frogs (right) were electrophoresed, transferred, immunoblotted, and exposed in parallel. [file peerj-02-558-s006.png]

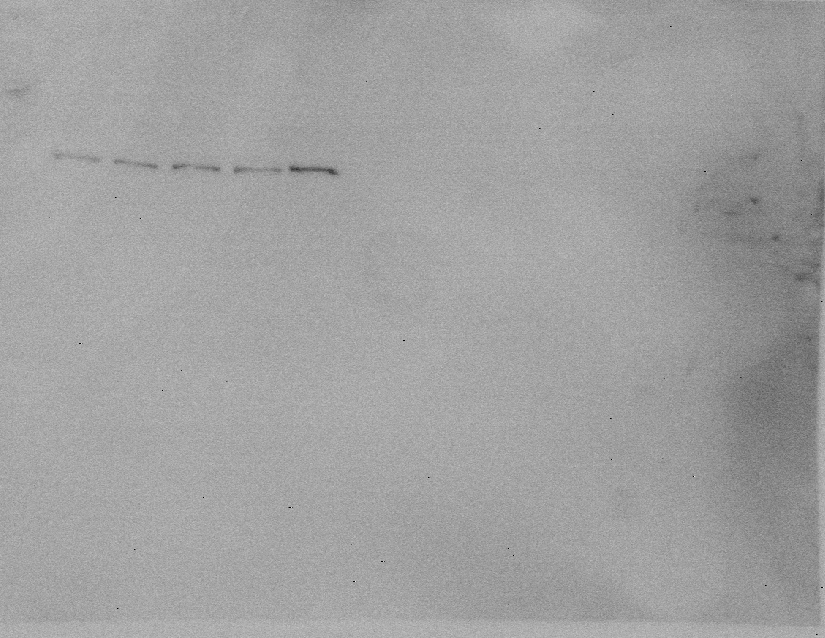

Supplement: Supplemental Information 7 — A full blot is presented here for liver, but in other instances PVDF membranes were typically cut at the approximate molecular weight of PKC so that membranes could be used for multiple antibodies (as described in Materials and Methods). 5 independently-prepared protein extracts from control frogs (left) and 5 independently-prepared protein extracts from frozen frogs (right) were electrophoresed, transferred, immunoblotted, and exposed in parallel. [file peerj-02-558-s007.png]

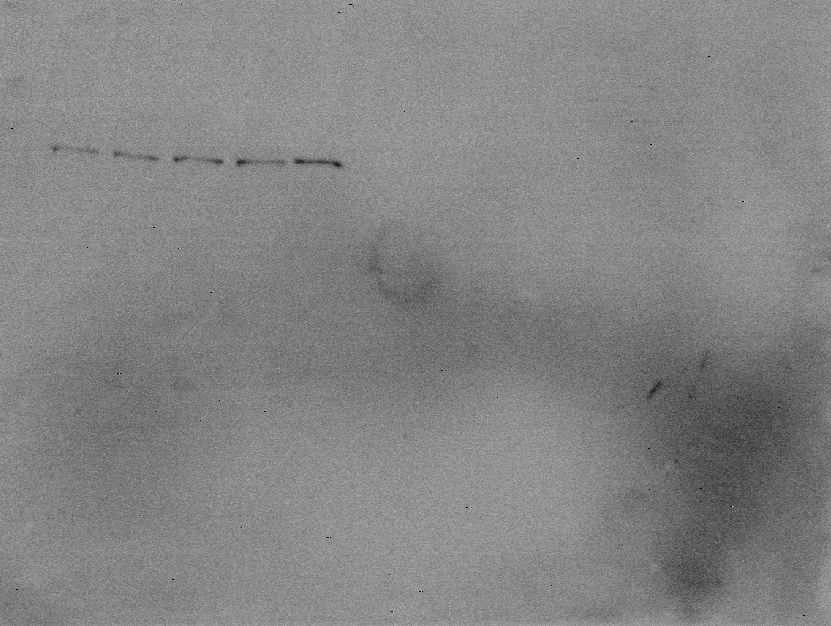

Supplement: Supplemental Information 8 — A full blot is presented here for liver, but in other instances PVDF membranes were typically cut at the approximate molecular weight of PKC so that membranes could be used for multiple antibodies (as described in Materials and Methods). 5 independently-prepared protein extracts from control frogs (left) and 5 independently-prepared protein extracts from frozen frogs (right) were electrophoresed, transferred, immunoblotted, and exposed in parallel. [file peerj-02-558-s008.png]

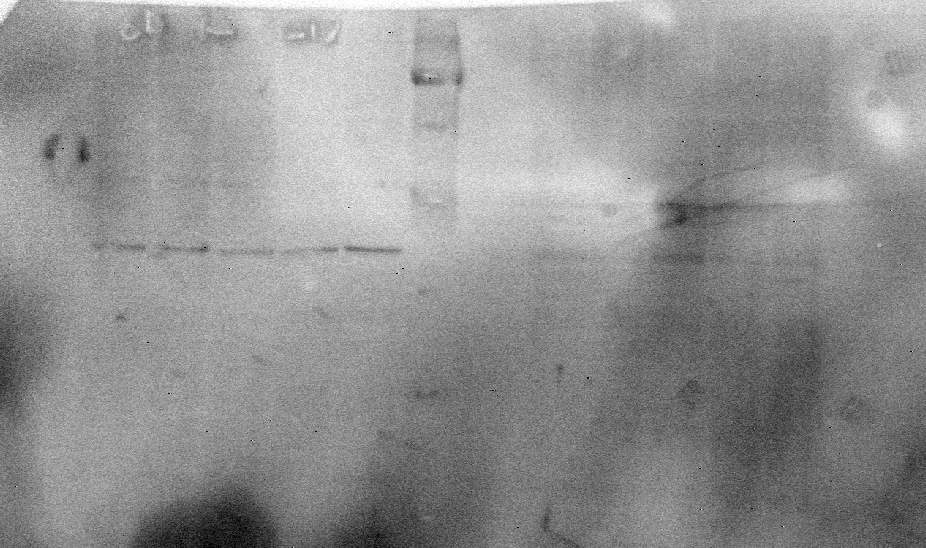

Supplement: Supplemental Information 9 — A full blot is presented here for liver, but in other instances PVDF membranes were typically cut at the approximate molecular weight of PKC so that membranes could be used for multiple antibodies (as described in Materials and Methods). 5 independently-prepared protein extracts from control frogs (left) and 5 independently-prepared protein extracts from frozen frogs (right) were electrophoresed, transferred, immunoblotted, and exposed in parallel. [file peerj-02-558-s009.png]
